# Obesity as a Risk Factor for Prostate Cancer Mortality: A Systematic Review and Dose-Response Meta-Analysis of 280,199 Patients

**DOI:** 10.3390/cancers13164169

**Published:** 2021-08-19

**Authors:** Mario Rivera-Izquierdo, Javier Pérez de Rojas, Virginia Martínez-Ruiz, Beatriz Pérez-Gómez, María-José Sánchez, Khalid Saeed Khan, José Juan Jiménez-Moleón

**Affiliations:** 1 Department of Preventive Medicine and Public Health, University of Granada, 18016 Granada, Spain; javier.perez.rojas.sspa@juntadeandalucia.es (J.P.d.R.); virmruiz@ugr.es (V.M.-R.); mariajose.sanchez.easp@juntadeandalucia.es (M.-J.S.); profkkhan@ugr.es (K.S.K.); jjmoleon@ugr.es (J.J.J.-M.); 2 Instituto de Investigación Biosanitaria ibs.GRANADA, 18012 Granada, Spain; 3 Centro de Investigación Biomédica en Red de Epidemiología y Salud Pública (CIBERESP), 28029 Madrid, Spain; bperez@isciii.es; 4 National Centre for Epidemiology, Department of Epidemiology of Chronic Diseases, Instituto de Salud Carlos III, 28029 Madrid, Spain; 5 Escuela Andaluza de Salud Pública (EASP), 18011 Granada, Spain

**Keywords:** body mass index, prostate cancer specific mortality, all-cause mortality, outcomes, causation

## Abstract

**Simple Summary:**

Results from individual studies on the association between obesity and prostate cancer mortality remain inconclusive; additionally, several large cohort studies have recently been conducted. We aimed to systematically review all available evidence and synthetize it using meta-analytic techniques. The results of our study showed that obesity was associated with prostate cancer specific mortality and all-cause mortality. The temporal association was consistent with a dose-response relationship. Our results demonstrated that obesity, a potentially modifiable prognostic factor, was associated with higher prostate cancer mortality. This study improved the evidence regarding the potential impact of lifestyle on improving prostate cancer prognosis. Strategies aimed at maintaining normal, or reducing abnormal, body mass index in diagnosed prostate cancer patients might improve survival. These results should guide urologists, oncologists, patients, policy-makers and primary care providers with respect to evidence-based practice and counselling concerning lifestyle changes after prostate cancer diagnosis.

**Abstract:**

The aim of this study was to systematically review all evidence evaluating obesity as a prognostic factor for PC mortality. Cohort and case-control studies reporting mortality among PC patients stratified by body mass index (BMI) were included. The risk of mortality among obese patients (BMI ≥ 30) was compared with the risk for normal weight (BMI < 25) patients, pooling individual hazard ratios (HR) in random-effects meta-analyses. Reasons for heterogeneity were assessed in subgroup analyses. Dose-response associations for BMI per 5 kg/m^2^ change were assessed. Among 7278 citations, 59 studies (280,199 patients) met inclusion criteria. Obesity was associated with increased PC-specific mortality (HR: 1.19, 95% CI: 1.10–1.28, I^2^: 44.4%) and all-cause mortality (HR: 1.09, 95% CI: 1.00–1.18, I^2^: 43.9%). There was a 9% increase (95% CI: 5–12%, I^2^: 39.4%) in PC-specific mortality and 3% increase (95% CI: 1–5%, I^2^: 24.3%) in all-cause mortality per 5 kg/m^2^ increase in BMI. In analyses restricted to the higher quality subgroup (NOS ≥ 8), obesity was associated with increased PC-specific mortality (HR: 1.24, 95% CI: 1.14–1.35, I^2^: 0.0%) and maintained the dose-response relationship (HR: 1.11 per 5 kg/m^2^ increase in BMI, 95% CI: 1.07–1.15, I^2^: 26.6%). Obesity had a moderate, consistent, temporal, and dose-response association with PC mortality. Weight control programs may have a role in improving PC survival.

## 1. Introduction

Prostate cancer (PC), the second most common cancer and the third leading cause of cancer death in men [1], is steadily increasing in incidence [2]. Worldwide, over 650 million adults are obese [3], and therefore exposed to the second most common cause of preventable death [4], while obesity has been proposed as a risk factor for aggressive PC [5]. Recent large studies, however, showed that this relationship is unclear [6,7]. The other known factors associated with PC mortality, older age, family history of any cancer and ethnicity [5], are not changeable. As a potentially modifiable factor, obesity merits evaluation as a prognostic factor.

Individual studies on the association between obesity and prostate cancer (PC) mortality show inconsistent results, including both positive [6] and negative [8] association. Evidence syntheses on the association between obesity and PC outcomes [9,10,11], when judged by AMSTAR 2 [12], demonstrate weaknesses in the description of the study population, investigation of the causes of heterogeneity, evaluation of the impact of risk of bias in stratified results, and reporting of funding or conflicts of interest. Since the last meta-analysis [9], 15 prognostic studies have been published [2,7,8,13,14,15,16,17,18,19,20,21,22,23,24] with data from 186,802 new PC patients added to the total. Consequently, the last review [9] could access only a third of the current body of evidence. Importantly, previous evidence syntheses have not formally evaluated causation [25]. Thus, there is need for a robust and reliable evaluation of the association between obesity and prostate cancer specific mortality (PCSM) and all-cause mortality (ACM) in patients diagnosed with PC.

We systematically reviewed all observational evidence that examined whether obesity influences mortality of PC patients and formally evaluated the dose-response relationship using meta-analytic techniques.

## 2. Materials and Methods

We used a prospective protocol registered in PROSPERO (CRD420202146000) [26]. The review team was composed of methodologists, investigators and researchers from public health, epidemiology, and urology specialties. For reporting, both Meta-analysis of Observational Studies in Epidemiology (MOOSE) [27] and the 2020 update of Preferred Reporting Items for Systematic Reviews and Meta-analyses (PRISMA) [28] guidelines (Table S1) were followed.

### 2.1. Search Strategy and Study Selection Criteria

We searched Medline, Web of Science and Scopus from database inception prior to April 1, 2021, with no language restrictions. We used the following search terms: “prostat* cancer”, “prostat* neoplasm” or “prostat* tumor” to search the population and combined it with relevant terms to the outcome “mortality”, “death”, “prognos*” or “survival” and exposure “obes*” or “body mass index” or “BMI” or “weight” (Table S2). We included studies with obesity (BMI ≥ 30) or BMI as continuous variable as exposure and mortality in PC patients as outcome (either PCSM, ACM or both) evaluated through observational analytical design (cohort and case-control). When the same cohort was reported more than once, we only considered the most recent study with the largest sample size for quantitative analyses. We excluded studies that assessed the risk of obesity on PC diagnosis with no prognostic evaluation or that did not provide sufficient data on mortality, as well as abstracts, case reports, reviews, and animal studies. When reported data were insufficient, we contacted the authors to include all available information. Studies reporting BMI only as a continuous variable were included in the synthesis. After electronic searches, we performed a manual search based on the reference lists from the selected studies and relevant reviews. Two independent reviewers (M.R.-I. and J.P.d.R.) conducted the search by screening titles and abstracts. Full text of potentially eligible studies was also assessed by two reviewers, and relevant information was retrieved. Potential disagreements were discussed and resolved with a third reviewer (J.J.J-M.).

### 2.2. Data Extraction and Quality Assessment

We used predesigned data extraction forms to collect information on authors, year of publication, study setting and design, population, median follow-up, exposures, outcomes, type of analysis and presence of conflicts of interests within all the selected studies. When a citation or article was written in a language different to English or Spanish, the evaluation involved the input of colleagues competent in that language. The methodological quality of the studies was evaluated independently by two researchers (MRI and JPD). Using the nine-star Newcastle-Ottawa Scale (NOS) [29], risk of bias regarding selection, comparability, and outcome (for cohort studies) or exposure (for case-control studies) were formally evaluated. Scores of 8 or more stars were considered as low risk of bias (high quality), and 6 to 7 stars were considered as having medium risk of bias. Discrepancies were solved by discussion with a senior reviewer (J.J.J.-M.) and consensus of all authors.

### 2.3. Exposure and Outcomes

Our main variable of exposure was obesity, either measured as body-mass-index equal or greater than 30 (BMI ≥ 30), compared with normal weight (BMI < 25) [30], or included as continuous BMI per 5 kg/m^2^ for dose-response analysis. Timing of measurement of exposure was divided in two groups: studies that measured BMI before or after the diagnosis. Waist circumference, waist-to-hip ratio and weight change were also extracted, however, given the small number of studies (<5), these exposures were not synthesized. The outcomes explored were PCSM and ACM.

### 2.4. Data Synthesis

As the outcome was relatively rare, the odds ratios (OR) and relative risks (RR) were considered as approximations of hazard ratios (HR), as recommended in previous analyses [9]. Forest plots were generated using PC patient survival data (HR and 95% CI) from the selected studies, as commonly reported in prognostic meta-analyses [9,31]. If a study provided risk estimates for PCSM and other-cause deaths, a risk estimate for ACM was calculated. If a study did not provide a summary estimate for the cohort and only reported estimations of subgroups of populations, the study was not included in the analysis. Normal weight (BMI < 25) represented the reference category for all the comparisons except when BMI was analyzed as a continuous variable.

We reported pooled HR and 95% CIs using a random-effects model to allow for unexplained heterogeneity across studies [32]. Heterogeneity was assessed through Q heterogeneity tests and I^2^ statistic. We compared the odds of prostate cancer specific mortality and all-cause mortality in the following groups: obesity (BMI ≥ 30) versus normal weight, as primary result, and abnormal weight (BMI ≥ 25) or overweight (BMI ≥ 25 and <30) versus normal weight as secondary outcomes. We also assessed association by an increase of 5 kg/m^2^ in BMI as the quantitative variable for dose-response association. When the study did not provide this estimation, it was calculated using the method of Greenland and Longnecker [33].

We undertook subgroup analyses planned a priori to detect differences based on the following factors: study quality according to NOS, level of the evidence according to the Quality Rating Schemes for Studies and Other Evidence, modified from the Oxford Centre for Evidence-based Medicine for ratings of individual studies [34], country, country development according to the International Monetary Fund, sample size, year of publication, moment of BMI measurement (before or after diagnosis), population setting, stage and treatment. Temporality was established in cohort studies that properly measured BMI according to NOS (directly measured by the researchers or collected from clinical histories or databases), around the time of PC diagnosis and conducted a median or mean follow-up ≥ 5 years (high quality according to NOS evaluation). The association of overweight (BMI ≥ 25 and <30) and abnormal weight (BMI ≥ 25) compared with normal weight (BMI < 25) with PCSM, and ACM was assessed as secondary analyses. Sensitivity analysis was performed by excluding studies that presented high risk of bias in the subgroup analyses. Funnel plots for potential publication bias and small-study effect were evaluated. Asymmetry was assessed using Egger’s regression test [35]. All statistical tests were 2-sided using a significance level of *p*  <  0.05. All analyses were performed using the Review Manager ^®^ from Cochrane Library and Stata (StataCorp^®^), version 15.0 (StataCorp. 2017. Stata Statistical Software: Release 15. StataCorp LLC, College Station, TX, USA).

## 3. Results

**Figure 1:**
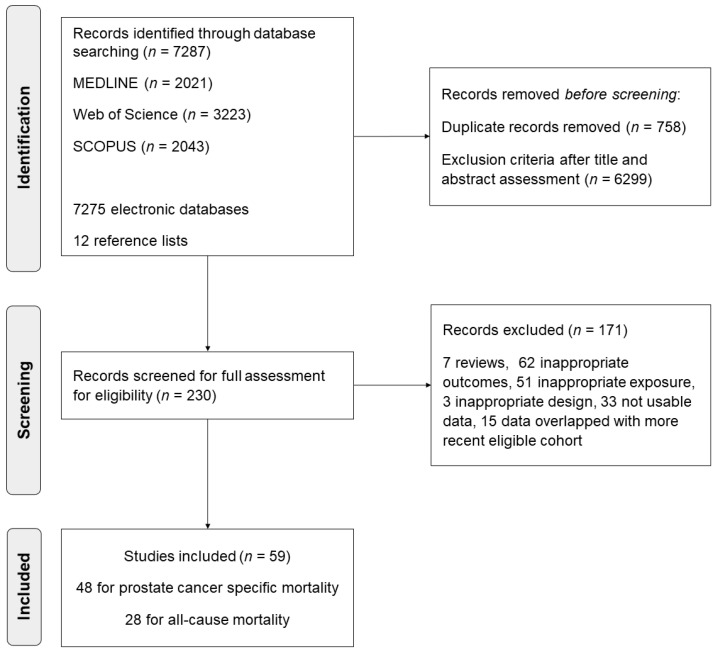
Study selection process in the systematic review and meta-analysis.

Of the 7278 citations identified, we selected 107 abstracts for detailed eligibility assessment (Figure 1). A total of 59 analyses reported in 57 published articles including 5146,333 participants and 280,199 PC patients met our inclusion criteria [2,7,8,13,14,15,16,17,18,19,20,21,22,23,24,36,37,38,39,40,41,42,43,44,45,46,47,48,49,50,51,52,53,54,55,56,57,58,59,60,61,62,63,64,65,66,67,68,69,70,71,72,73,74,75,76,77]. Sample sizes ranged from 55 to 90,694 PC patients, with a median of 1442. Among them, 48 studies (81.4%) provided data on PCSM, 28 studies (47.5%) provided data on ACM, and 17 studies (28.8%) studies provided data on both outcomes.

Of the 59 primary studies, 1 was a retrospective case-control study (1.7%) and 58 were cohort studies, of which 39 were prospective (67.2%) and 19 retrospective (32.8%). Data were obtained from population-based incident PC cohorts in 27 studies (45.8%), cohorts of incident PC among industry workers in 3 studies (5.1%), cohorts of patients after radical prostatectomy in 8 studies (13.6%), studies of patients with localized PC diagnosis in 9 cohorts (15.3%) and of patients with advanced PC diagnosis in 10 cohorts (17.0%). One study was conducted in African-Caribbean ancestry patients and 1 study was conducted in PC patients receiving androgen-deprivation therapy. Only 5 studies (8.5%) reported financial interests or potential conflicts of interest. Regarding the exposure of interest, its operational definition varied among the selected studies. Therefore, 38 studies (64.4%) used the World Health Organization categories, considering overweight as BMI ≥ 25 and < 30; and obesity as BMI ≥ 30, 15 studies (25.4%) used different categories and 6 studies (10.2%) only considered BMI as continuous variable. Seventeen (28.8%) of the studies were published in 2016 or later. BMI before diagnosis was assessed in 28 studies (47.5%), while BMI after diagnosis was assessed in 32 studies (54.2%). Detailed information on the 59 primary studies is available in Table S3.

**Figure 2:**
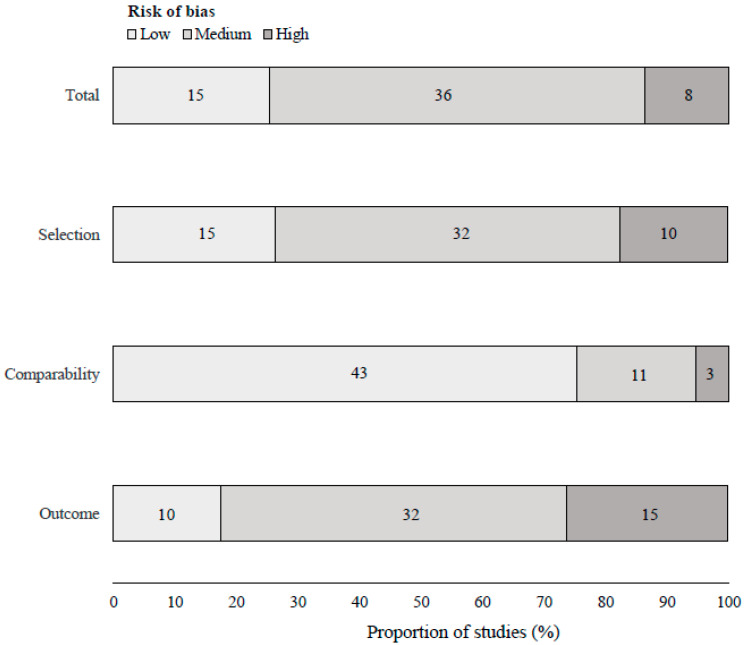
Newcastle-Ottawa Scale assessment of the cohort studies analyzing the association between obesity and mortality.

**Figure 3:**
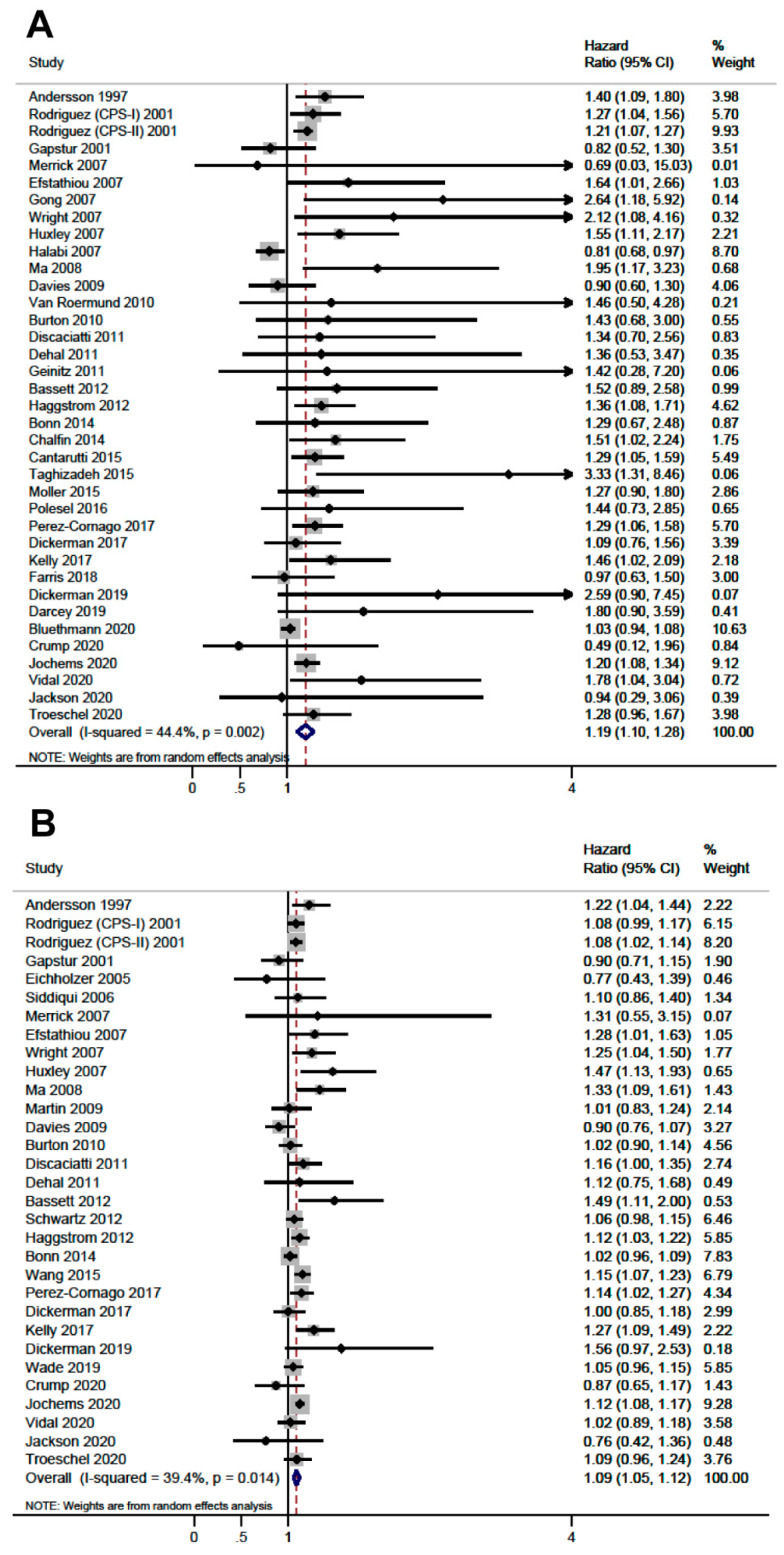
Forest plots of the association for prostate cancer specific mortality. (**A**) Obesity (body mass index ≥ 30 kg/m^2^) compared to normal weight (body mass index < 25 kg/m^2^). (**B**) Continuous body mass index per 5 kg/m^2^ increment.

**Figure 4:**
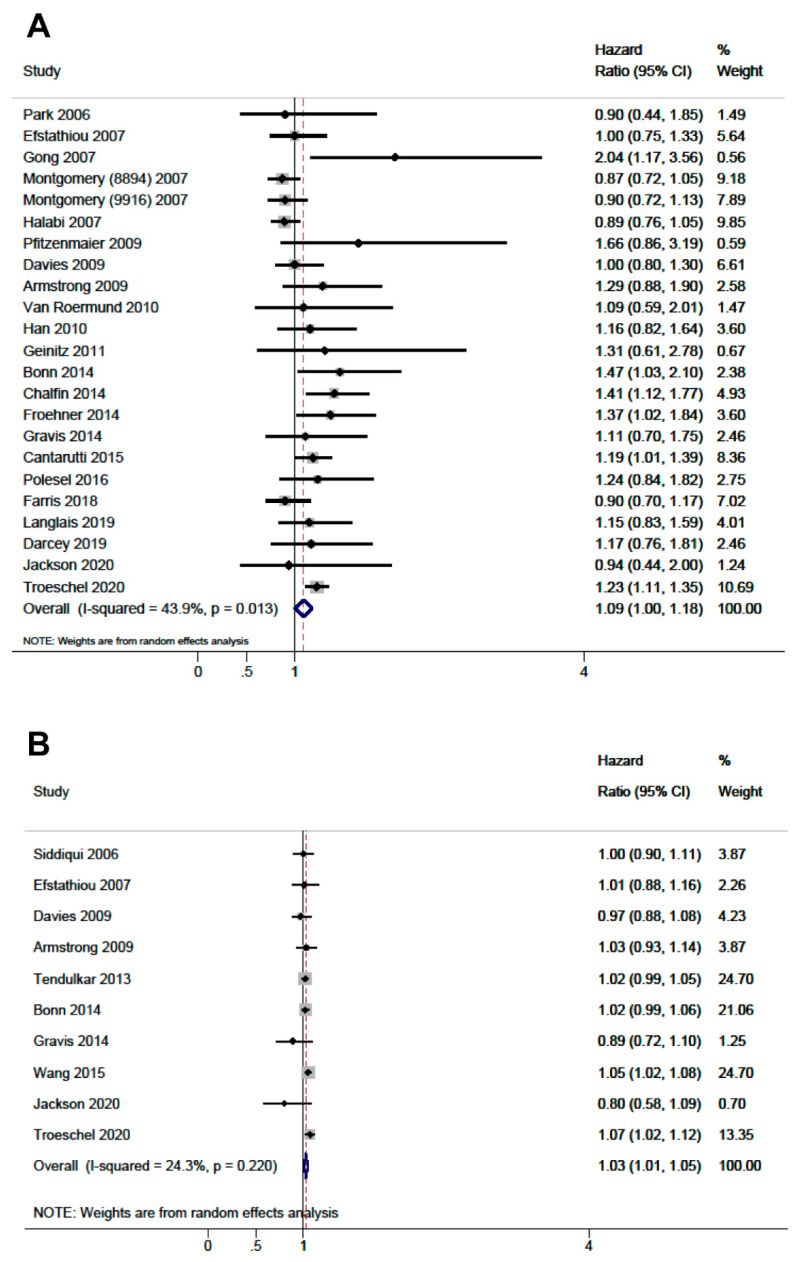
Forest plots of the association for all-cause mortality. (**A**) Obesity (body mass index ≥ 30 kg/m^2^) compared to normal weight (body mass index < 25 kg/m^2^). (**B**) Continuous body mass index per 5 kg/m^2^ increment.

The study quality captured by NOS regarding risk of bias in study selection, comparability of the cohorts and outcome assessment is summarized in Figure 2 and detailed in Table S4. The mean NOS score was 6.8 (median 7, range 3–9), 15 (25.4%) studies presented low risk of bias (8–9 stars) according to NOS, 36 (61.0%) studies presented medium risk of bias and 8 studies (13.6%) presented high risk of bias. The pooled associations of obesity, compared with normal weight, and continuous BMI with PCSM and with ACM are presented in Figure 3 and Figure 4, respectively.

Obesity was associated with a greater hazard of PCSM (HR: 1.19, CI 95%: 1.10–1.28, I^2^:44.4%) and ACM (HR: 1.09, CI 95%: 1.00–1.18, I^2^:43.9%). A similar result was observed when we evaluated BMI as continuous variable, either with PCSM (HR per 5 kg/m^2^: 1.09, CI 95%: 1.05–1.12, I^2^: 39.4%) or with ACM (HR per 5 kg/m^2^: 1.03, CI 95%: 1.01–1.05, I^2^: 24.3%), suggesting a dose-response relationship. Table 1 presents the subgroup analyses based on population, stage, treatment, country, country status, quality of the evidence, risk of bias, causal criteria (detailed in Table S5), exposure measurement, and year of publication, and showed no significant differences between BMI strata. Heterogeneity was considerably reduced when stratified by population of origin and country, and when we restricted the analysis to high-quality studies according to NOS. Prospective cohort studies (HR: 1.19, 95% CI: 1.10–1.28, I^2^:34.4%), high-quality studies according to NOS (HR: 1.24, 95% CI: 1.14–1.35, I^2^:0.0%) and population-based cohorts (HR: 1.24, 95% CI: 1.18–1.31, I^2^:0.0%) consistently showed a relationship between obesity and PCSM (Table 1). Regarding ACM, studies with higher quality according to NOS (8–9 stars) showed a positive association with obesity (HR: 1.46, 95% CI: 1.01–1.91, I^2^:7.3%). The sensitivity analysis excluding lower quality studies did not show differences in the estimations of any comparison. The timing of measurements of BMI (prediagnosis or postdiagnosis) was a source of heterogeneity. Studies using prediagnosis BMI showed association between obesity and PCSM (HR: 1.23, 95% CI: 1.17–1.30, I^2^:0.0%), but not with ACM (HR: 1.09, 95% CI: 0.98–1.18, I^2^: 47.4%). In contrast, for postdiagnosis BMI, obesity was associated with ACM (HR: 1.20, 95% CI: 1.03–1.37, I^2^:0.0%), but not with PCSM (HR. 1.07, 95% CI: 1.00–1.14, I^2^: 48.5%). Studies that adjusted for cancer stage showed estimates of association similar to those that did not. The association of overweight and abnormal weight, assessed as secondary analyses, with PCSM and ACM is shown in Table S6. We did not observe evidence of small-studies effect for the analyzed outcomes in funnel plot analysis, except for obesity and PCSM where the funnel was truncated with small studies showing positive association missing (*p*-value of Egger test = 0.005; Table S7.

## 4. Discussion

In this meta-analysis, compiling all available data for precise quantitative estimation of the prognostic effect of obesity in PC mortality, we found that BMI ≥ 30 was associated with PCSM and ACM compared with normal weight. Both mortality outcomes showed dose-response relationship with every 5 kg/m^2^ unit increase in BMI. In higher quality prospective studies evaluating temporal association, BMI ≥ 30 was associated with increased PCSM and showed dose-response association.

We performed a comprehensive literature search without language restrictions, increasing our potential to capture all relevant studies. Owing to the large sample size, we were able to undertake powerful analyses, including predefined subgroup analyses, to generate reliable results. There was considerable heterogeneity in the pooled analyses, and we used random effects models to obtain conservative precision estimates. The statistical significance of the observed heterogeneity could reflect the large number of studies we captured [78]. The exploration of reasons for heterogeneity showed that the main findings were not sensitive to variations in subgroups based on populations, settings, disease stage, and interventions. The measurement of exposure before or after the diagnosis provides a dichotomized assessment of a wide time range. The results in the postdiagnosis exposure measurement subgroup confirmed the prognostic association of continuous BMI with PCSM, which contributes to the specificity element of the causal criteria [79], and with ACM, consistent with the general adverse effects of obesity on overall survival. Conversely, obesity exposure throughout life captured in prediagnosis measurement showed an association with PCSM, although no association was found for ACM. The association between BMI and PC mortality might be different according to the treatment (e.g., better surgical success in patients with normal weight treated with radical prostatectomy). Subgroup analyses by ethnicity and other potentially important factors was not possible given that most of the selected studies did not report stratified results. However, adjusted hazard ratios were considered in the pooled analyses (Table S3) to reduce residual confusion. Our main findings were backed by the high-quality subgroup of studies, highlighting that the observed association of obesity with PC prognosis merits consideration.

The assessment of causation is integral to the evaluation of findings of observational meta-analyses [79]. We evaluated whether our observed association fulfilled the classical Bradford Hill principles of causation [25]. Our assessment showed evidence of moderate strength of association, consistency, temporality, specificity, dose-response gradient, biological plausibility and analogy (Table S5). The association measured by pooled HR was statistically significant overall. The HR point estimate showed an increased strength of association in the higher-quality subgroup of studies. Consistency of individual studies, analyzed graphically, showed that point estimates of individual HRs on over three-quarters of the studies had an association. Although statistically I^2^ measurements showed variation, this reflected differences in size of the association observed rather than differences in its direction. The association within subgroups showed lower level of heterogeneity. The association was consistently observed across the subgroups including different stages, treatments (e.g., prostatectomy or androgen deprivation therapy) and populations of PC patients. Studies that analyzed obesity with measurements different from BMI also showed consistent association with PC mortality [80,81,82]. Temporality was established by longitudinal (cohort) studies. The specificity of the association was reflected in the results concerning PCSM. Moreover, the studies synthetized in our meta-analysis were mostly adjusted by several potential confounders, as shown in Table S3. Regarding biological gradient, we showed dose-response relationship by using continuous BMI as exposure, which was associated with PCSM and ACM. The biological plausibility of the association is underpinned by several postulated mechanisms explaining the relationship between BMI and PC death [9]. For instance, obesity is the most common cause of insulin resistance, which has been associated with a greater inflammatory state, a risk factor for cancer progression [83]. Also, molecular mechanisms connecting obesity with PC and other urothelial cancers have been broadly established [84]. Finally, the relationship with PC outcomes met analogy criterion, as obesity has been linked for the last three decades to mortality from numerous types of cancer [11,85] and to other outcomes related to PC, for instance, the presence of metastases [86]. Therefore, objectively, several criteria for causation were met.

The World Cancer Research Fund [87] reported an increased risk of being diagnosed with advanced PC in obese patients, although large studies have recently questioned this point [6,7]. Our findings provide strong evidence that obese men diagnosed with PC are more likely to have a worse prognosis. Not only does our review strengthen the prevailing hypothesis concerning the association between prediagnosis obesity and PCSM [9,11,80,81,82], it suggests an impact of postdiagnosis obesity on PC mortality outcomes. As it is potentially modifiable by lifestyle changes, future evaluations of the role of weight loss among obese patients with PC are required. For example, randomized interventions on diet and physical activity are needed to analyze PC outcomes [88,89]. Guidelines and patient information documents concerning PC would need to be updated to emphasize the role of obesity in prognosis.

## 5. Conclusions

Obesity currently poses an alarming burden on individuals, societies, and economies. Our study shows that in PC patients, obesity, a potentially modifiable risk factor, is moderately associated with temporality and a dose-response with PCSM and ACM. Therefore, obesity increases mortality in prostate cancer patients, according to the current observational evidence. This information should be useful in counselling PC patients and in planning future research concerning their lifestyle.

## Figures and Tables

**Table 1:** Subgroup analysis of the pooled association of body mass index with prostate cancer specific mortality and all-cause mortality.

Subgroup	Prostate Cancer Specific Mortality						All-Cause Mortality					
	Obesity (BMI ≥ 30) Compared with Normal Weight (BMI < 25)			BMI Continuous Per 5 kg/m^2^			Obesity (BMI > 30) Compared with Normal Weight (BMI < 25)			BMI Continuous Per 5 kg/m^2^		
	N	HR (95% CI)	I^2^	N	HR (95% CI)	I^2^	N	HR (95% CI)	I^2^	N	HR (95% CI)	I^2^
**All Studies (Total)**	37	1.19 (1.10–1.28)	44.3	31	1.09 (1.05–1.12)	44.3	23	1.09 (1.00–1.18)	43.9	10	1.03 (1.01–1.05)	24.3
**Population**												
Population-Based Incident PC	22	1.24 (1.18–1.31)	0.0	18	1.10 (1.07–1.14)	31.3	6	1.10 (0.92–1.28)	25.5	0	-	-
Industry Workers Incident PC	2	1.12 (0.55–1.68)	78.5	3	1.01 (0.74–1.27)	65.9	0	-	-	0	-	-
Radical Prostatectomy	2	1.58 (1.06–2.10)	0.0	2	1.04 (0.91–1.17)	0.0	5	1.29 (1.11–1.48)	0.0	1	1.00 (0.89–1.11)	-
Localized PC	8	1.04 (0.97–1.10)	0.0	6	1.05 (0.97–1.12)	55.3	5	1.20 (1.09–1.30)	0.0	4	1.04 (1.01–1.07)	39.3
Advanced PC	2	1.12 (0.33–1.91)	73.3	1	1.28 (0.97–1.59)	-	6	0.91 (0.83–1.00)	0.0	4	1.02 (0.99–1.05)	0.0
African-Caribbean Ancestry	1	0.94 (0.44–2.33)	-	1	0.76 (0.29–1.23)	-	1	0.94 (0.16–1.72)	-	1	0.80 (0.55–1.05)	-
**Country**												
USA	17	1.13 (1.00–1.28)	61.6	15	1.09 (1.04–1.14)	36.8	12	1.05 (0.93–1.17)	63.8	7	1.04 (1.02–1.05)	0.0
European Nordic Countries	9	1.22 (1.12–1.32)	0.0	9	1.08 (1.01–1.14)	45.5	2	1.22 (1.04–1.40)	0.0	1	1.02 (0.98–1.05)	-
European Central Countries	7	1.33 (1.14–1.53)	0.0	4	1.09 (1.01–1.17)	25.2	6	1.26 (1.02–1.50)	0.0	1	0.89 (0.70–1.08)	-
Other	4	1.51 (1.11–1.92)	0.0	3	1.25 (0.80–1.70)	68.9	3	1.04 (0.67–1.41)	0.0	1	0.80 (0.55–1.05)	-
**Country status**												
Developed Countries	35	1.18 (1.10–1.27)	45.2	29	1.09 (1.05–1.12)	36.6	22	1.09 (1.00–1.19)	46.3	9	1.03 (1.01–1.05)	8.4
Developing Countries	2	1.47 (0.98–1.97)	0.0	2	1.13 (0.43–1.82)	80.3	1	0.94 (0.44–2.00)	-	1	0.80 (0.55–1.05)	-
**Exposure Measurement** ^1^												
Prediagnosis BMI	22	1.23 (1.17–1.30)	0.0	21	1.09 (1.06–1.13)	35.0	19	1.08 (0.98–1.18)	47.4	0	-	-
Postdiagnosis BMI	15	1.10 (0.96–1.23)	42.8	10	1.07 (1.00–1.14)	48.5	4	1.20 (1.03–1.37)	0.0	10	1.03 (1.01–1.05)	24.3
**Quality of the Evidence** ^2^												
Level 2 (Prospective Cohort)	26	1.19 (1.10–1.28)	34.4	26	1.09 (1.05−1.13)	35.1	12	1.04 (0.92−1.16)	50.3	6	1.02 (0.98−1.07)	20.5
Level 3	11	1.27 (0.99−1.55)	57.5	5	1.08 (0.99−1.17)	61.5	11	1.17 (1.02−1.32)	40.0	4	1.03 (1.00−1.06)	46.2
**Risk of Bias**												
NOS: 8−9	9	1.24 (1.14−1.35)	0.0	12	1.11 (1.07−1.15)	26.6	2	1.46 (1.01−1.91)	7.3	1	1.05 (1.02−1.08)	-
NOS: 6−7	27	1.17 (1.07−1.27)	49.2	18	1.07 (1.02−1.12)	41.6	16	1.10 (1.01−1.20)	36.9	6	1.02 (0.98−1.06)	34.2
NOS <6	1	0.69 (0.03−15.03)	-	1	1.31 (0.55−3.15)	-	5	0.90 (0.80−1.07)	10.3	3	1.02 (0.99−1.05)	0.0
**Design**												
Cohort	36	1.19 (1.11–1.28)	45.9	30	1.09 (1.05–1.12)	39.1	22	1.09 (1.00–1.18)	43.9	9	1.03 (1.01–1.05)	8.4
Case-Control	1	0.94 (0.44–2.33)	-	1	0.79 (0.29–1.23)	-	1	0.94 (0.16–1.72)	-	1	0.80 (0.55–1.05)	-
**Stage**												
Adjustment for Stage	13	1.11 (0.95–1.27)	44.0	8	1.08 (1.01–1.16)	52.1	14	1.08 (0.95–1.21)	55.6	5	1.04 (1.01–1.07)	39.8
Not Adjustment for Stage	24	1.22 (1.16–1.29)	0.0	23	1.09 (1.05–1.13)	35.8	9	1.12 (1.00–1.23)	10.0	5	1.01 (0.99–1.04)	0.0
**Year of Publication**												
<2016	24	1.19 (1.10–1.28)	50.1	21	1.09 (1.05–1.12)	39.4	6	1.13 (0.99–1.27)	22.1	2	0.97 (0.71–1.22)	75.9
≥2016	13	1.15 (1.04–1.26)	28.9	10	1.08 (1.03–1.14)	35.4	17	1.08 (0.97–1.19)	42.4	8	1.03 (1.01–1.04)	0.0

BMI, body mass index; HR, hazard ratio; NOS, Newcastle-Ottawa scale; PC, prostate cancer. *p*-values of the table show the results from heterogeneity analyses of each subgroup. ^1^ Of the 28 studies evaluating prediagnosis BMI, 1 collected BMI one year before diagnosis [67], 1 measured BMI at 18 years old [14], and 26 collected BMI from retrospective sources or at recruitment and time from measurement to diagnosis was unreported. ^2^ Quality of the evidence according to the Quality Rating Schemes for Studies and Other Evidence, modified from the Oxford Centre for Evidence-based Medicine for ratings of individual studies [32].

## Data Availability

All data generated or analyzed during this study are included in the manuscript and its Supporting Information files.

## Supplementary Materials

The following are available online at https://www.mdpi.com/article/10.3390/cancers13164169/s1, Table S1: PRISMA checklist of the systematic review and meta-analysis, Table S2: Search strategy, Table S3: Main characteristics of the studies included in the systematic review of prognosis of prostate cancer with respect to obesity, Table S4: Methodological quality assessment of the selected studies according to Newcastle-Ottawa Scale, Table S5: Assessment of potential causation criteria for the association assessed in this systematic review, Table S6: Forest-plot of the associations between obesity (BMI ≥ 30), overweight (BMI < 30 and ≥25) and abnormal weight (BMI ≥ 25), compared to normal weight (BMI < 25), and continuous BMI per 5 units, on prostate cancer specific mortality and all-cause mortality, Table S7: Funnel plots of the pooled associations between obesity (BMI ≥ 30) and continuous BMI per 5 kg/m^2^ with prostate cancer specific mortality and all-cause mortality.

## References

[1] Sung H., Ferlay J., Siegel R.L., Laversanne M., Soerjomataram I., Jemal A., Bray F. (2021). Global cancer statistics 2020: GLOBOCAN estimates of incidence and mortality worldwide for 36 cancers in 185 countries. CA Cancer J. Clin..

[2] Fillon M. (2020). Rates of advanced prostate cancer continue to increase. CA Cancer J. Clin..

[3] World Health Organization Obesity and Overweight. https://www.who.int/news-room/fact-sheets/detail/obesity-and-overweight.

[4] Panuganti K.K., Nguyen M., Kshirsagar R.K. (2020). Obesity. StatPearls.

[5] Schatten H. (2018). Brief Overview of Prostate Cancer Statistics, Grading, Diagnosis and Treatment Strategies. Adv. Exp. Med. Biol..

[6] Genkinger J.M., Wu K., Wang M., Albanes D., Black A., van den Brandt P.A., Burke K.A., Cook M.B., Gapstur S.M., Giles G.G. (2020). Measures of body fatness and height in early and mid-to-late adulthood and prostate cancer: Risk and mortality in The Pooling Project of Prospective Studies of Diet and Cancer. Ann. Oncol..

[7] Jochems S.H.J., Stattin P., Häggström C., Järvholm B., Orho-Melander M., Wood A.M., Stocks T. (2020). Height, body mass index and prostate cancer risk and mortality by way of detection and cancer risk category. Int. J. Cancer..

[8] Jackson M.D., Tulloch-Reid M.K., McCaw-Binns A.M., Aiken W., Ferguson T.S., Bennett N.R., Harrison L., Badaloo A., McGrowder D., Grindley A. (2020). Central adiposity at diagnosis may reduce prostate cancer-specific mortality in African-Caribbean men with prostate cancer: 10-year follow-up of participants in a case-control study. Cancer Causes Control..

[9] Zhong S., Yan X., Wu Y., Zhang X., Chen L., Tang J., Zhao J. (2016). Body mass index and mortality in prostate cancer patients: A dose-response meta-analysis. Prostate Cancer Prostatic Dis..

[10] Zhang X., Zhou G., Sun B., Zhao G., Liu D., Sun J., Liu C., Guo H. (2015). Impact of obesity upon prostate cancer-associated mortality: A meta-analysis of 17 cohort studies. Oncol. Lett..

[11] Cao Y., Ma J. (2011). Body mass index, prostate cancer-specific mortality, and biochemical recurrence: A systematic review and meta-analysis. Cancer Prev. Res..

[12] Shea B.J., Reeves B.C., Wells G., Thuku M., Hamel C., Moran J., Moher D., Tugwell P., Welch V., Kristjansson E. (2017). AMSTAR 2: A critical appraisal tool for systematic reviews that include randomised or non-randomised studies of healthcare interventions, or both. BMJ.

[13] Bluethmann S.M., Wang M., Wasserman E., Chen C., Zaorsky N.G., Hohl R.J., McDonald A.C. (2020). Prostate cancer in Pennsylvania: The role of older age at diagnosis, aggressiveness, and environmental risk factors on treatment and mortality using data from the Pennsylvania Cancer Registry. Cancer Med..

[14] Crump C., Stattin P., Brooks J.D., Stocks T., Sundquist J., Sieh W., Sundquist K. (2020). Early-Life Cardiorespiratory Fitness and Long-term Risk of Prostate Cancer. Cancer Epidemiol. Biomarkers Prev..

[15] Vidal A.C., Oyekunle T., Howard L.E., De Hoedt A.M., Kane C.J., Terris M.K., Cooperberg M.R., Amling C.L., Klaassen Z., Freedland S.J. (2020). Obesity, race, and long-term prostate cancer outcomes. Cancer.

[16] Troeschel A.N., Hartman T.J., Jacobs E.J., Stevens V.L., Gansler T., Flanders W.D., McCullough L.E., Wang Y. (2020). Postdiagnosis Body Mass Index, Weight Change, and Mortality From Prostate Cancer, Cardiovascular Disease, and All Causes Among Survivors of Nonmetastatic Prostate Cancer. J. Clin. Oncol..

[17] Langlais C.S., Cowan J.E., Neuhaus J., Kenfield S.A., Van Blarigan E.L., Broering J.M., Cooperberg M.R., Carroll P., Chan J.M. (2019). Obesity at Diagnosis and Prostate Cancer Prognosis and Recurrence Risk Following Primary Treatment by Radical Prostatectomy. Cancer Epidemiol. Biomarkers Prev..

[18] Darcey E., Pereira G., Salter A., Fritschi L., Leavy J., Ambrosini G.L., Boyle T. (2019). The Impact of Lifestyle-related Factors on Survival After a Prostate Cancer Diagnosis. Eur. Urol..

[19] Wade K.H., Carslake D., Tynelius P., Davey Smith G., Martin R.M. (2019). Variation of all-cause and cause-specific mortality with body mass index in one million Swedish parent-son pairs: An instrumental variable analysis. PLoS Med..

[20] Farris M.S., Courneya K.S., Kopciuk K.A., McGregor S.E., Friedenreich C.M. (2018). Anthropometric measurements and survival after a prostate cancer diagnosis. Br. J. Cancer.

[21] Hu M.B., Yang T., Hu J.M., Zhu W.H., Jiang H.W., Ding Q. (2018). Prognostic factors in Chinese patients with prostate cancer receiving primary androgen deprivation therapy: Validation of Japan Cancer of the Prostate Risk Assessment (J-CAPRA) score and impacts of pre-existing obesity and diabetes mellitus. Int. J. Clin. Oncol..

[22] Perez-Cornago A., Appleby P.N., Pischon T., Tsilidis K.K., Tjønneland A., Olsen A., Overvad K., Kaaks R., Kühn T., Boeing H. (2017). Tall height and obesity are associated with an increased risk of aggressive prostate cancer: Results from the EPIC cohort study. BMC Med..

[23] Dickerman B.A., Ahearn T.U., Giovannucci E., Stampfer M.J., Nguyen P.L., Mucci L.A., Wilson K.M. (2017). Weight change, obesity and risk of prostate cancer progression among men with clinically localized prostate cancer. Int. J. Cancer.

[24] Kelly S.P., Graubard B.I., Andreotti G., Younes N., Cleary S.D., Cook M.B. (2016). Prediagnostic Body Mass Index Trajectories in Relation to Prostate Cancer Incidence and Mortality in the PLCO Cancer Screening Trial. J. Natl. Cancer Inst..

[25] Hill A.B. (1965). The environment and disease: Association or causation?. Proc. R. Soc. Med..

[26] Rivera-Izquierdo M., Jiménez-Moleón J.J. Is Obesity A Prognostic Factor for Prostate Cancer? A Systematic Review and Meta-Analysis of Analytic Studies. PROSPERO: International Prospective Register of Systematic Reviews 2020: CRD42020214600. http://www.crd.york.ac.uk/PROSPERO/display_record.asp?ID=CRD42020214600.

[27] Stroup D.F., Berlin J.A., Morton S.C., Olkin I., Williamson G.D., Rennie D., Moher D., Becker B.J., Sipe T.A., Thacker S.B. (2000). Meta-analysis of observational studies in epidemiology: A proposal for reporting. Meta-analysis Of Observational Studies in Epidemiology (MOOSE) group. JAMA.

[28] Page M.J., McKenzie J.E., Bossuyt P.M., Boutron I., Hoffmann T.C., Mulrow C.D., Shamseer L., Tetzlaff J.M., Akl E.A., Brennan S.E. (2021). The PRISMA 2020 statement: An updated guideline for reporting systematic reviews. BMJ.

[29] Wells G.A., Shea B., O’Connell D., Peterson J., Welch V., Losos M., Tugwell P. The Newcastle-Ottawa Scale (NOS) for Assessing the Quality of Nonrandomised Studies in Meta-Analyses. http://www.ohri.ca/programs/clinical_epidemiology/oxford.asp.

[30] World Health Organization Body Mass Index. https://www.who.int/data/gho/data/themes/theme-details/GHO/body-mass-index-(bmi).

[31] Riley R.D., Moons K.G.M., Snell K.I.E., Ensor J., Hooft L., Altman D.G., Hayden J., Collins G.S., Debray T.P.A. (2019). A guide to systematic review and meta-analysis of prognostic factor studies. BMJ.

[32] Riley R.D., Higgins J.P., Deeks J.J. (2011). Interpretation of random effects metaanalyses. BMJ.

[33] Greenland S., Longnecker M.P. (1992). Methods for trend estimation from summarized dose-response data, with applications to meta-analysis. Am. J. Epidemiol..

[34] Centre for Evidence-Based Medicine Levels of Evidence. https://www.cebm.ox.ac.uk/resources/levels-of-evidence.

[35] Egger M., Davey Smith G., Schneider M., Minder C. (1997). Bias in meta-analysis detected by a simple, graphical test. BMJ.

[36] Polesel J., Gini A., Dal Maso L., Stocco C., Birri S., Taborelli M., Serraino D., Zucchetto A. (2016). The impact of diabetes and other metabolic disorders on prostate cancer prognosis. J. Diabetes Complicat..

[37] Cushen S.J., Power D.G., Murphy K.P., McDermott R., Griffin B.T., Lim M., Daly L., MacEneaney P., O’ Sullivan K., Prado C.M. (2016). Impact of body composition parameters on clinical outcomes in patients with metastatic castrate-resistant prostate cancer treated with docetaxel. Clin. Nutr. ESPEN.

[38] Fowke J.H., McLerran D.F., Gupta P.C., He J., Shu X.O., Ramadas K., Tsugane S., Inoue M., Tamakoshi A., Koh W.P. (2015). Associations of body mass index, smoking, and alcohol consumption with prostate cancer mortality in the Asia Cohort Consortium. Am. J. Epidemiol..

[39] Wang L.S., Murphy C.T., Ruth K., Zaorsky N.G., Smaldone M.C., Sobczak M.L., Kutikov A., Viterbo R., Horwitz E.M. (2015). Impact of obesity on outcomes after definitive dose-escalated intensity-modulated radiotherapy for localized prostate cancer. Cancer.

[40] Cantarutti A., Bonn S.E., Adami H.O., Grönberg H., Bellocco R., Bälter K. (2015). Body mass index and mortality in men with prostate cancer. Prostate.

[41] Taghizadeh N., Boezen H.M., Schouten J.P., Schröder C.P., Elisabeth de Vries E.G., Vonk J.M. (2015). BMI and lifetime changes in BMI and cancer mortality risk. PLoS ONE.

[42] Mohammed A.A., El-Tanni H., Ghanem H.M., Farooq M.U., El Saify A.M., Al-Zahrani A.S., El-Shentenawy A., El-Khatib H.M. (2015). Impact of body mass index on clinico-pathological parameters and outcome in patients with metastatic prostate cancer. J. Egypt. Natl. Canc. Inst..

[43] Møller H., Roswall N., Van Hemelrijck M., Larsen S.B., Cuzick J., Holmberg L., Overvad K., Tjønneland A. (2015). Prostate cancer incidence, clinical stage and survival in relation to obesity: A prospective cohort study in Denmark. Int. J. Cancer..

[44] Bonn S.E., Wiklund F., Sjölander A., Szulkin R., Stattin P., Holmberg E., Grönberg H., Bälter K. (2014). Body mass index and weight change in men with prostate cancer: Progression and mortality. Cancer Causes Control.

[45] Chalfin H.J., Lee S.B., Jeong B.C., Freedland S.J., Alai H., Feng Z., Trock B.J., Partin A.W., Humphreys E., Walsh P.C. (2014). Obesity and long-term survival after radical prostatectomy. J. Urol..

[46] Haque R., Van Den Eeden S.K., Wallner L.P., Richert-Boe K., Kallakury B., Wang R., Weinmann S. (2014). Association of body mass index and prostate cancer mortality. Obes. Res. Clin. Pract..

[47] Froehner M., Kellner A.E., Koch R., Baretton G.B., Hakenberg O.W., Wirth M.P. (2014). A combined index to classify prognostic comorbidity in candidates for radical prostatectomy. BMC Urol..

[48] Gravis G., Boher J.M., Fizazi K., Joly F., Priou F., Marino P., Latorzeff I., Delva R., Krakowski I., Laguerre B. (2015). Prognostic Factors for Survival in Noncastrate Metastatic Prostate Cancer: Validation of the Glass Model and Development of a Novel Simplified Prognostic Model. Eur. Urol..

[49] Tendulkar R.D., Hunter G.K., Reddy C.A., Stephans K.L., Ciezki J.P., Abdel-Wahab M., Stephenson A.J., Klein E.A., Mahadevan A., Kupelian P.A. (2013). Causes of mortality after dose-escalated radiation therapy and androgen deprivation for high-risk prostate cancer. Int. J. Radiat. Oncol. Biol. Phys..

[50] Bassett J.K., Severi G., Baglietto L., MacInnis R.J., Hoang H.N., Hopper J.L., English D.R., Giles G.G. (2012). Weight change and prostate cancer incidence and mortality. Int. J. Cancer.

[51] Schwartz G.G., Skinner H.G. (2012). A prospective study of total and ionized serum calcium and time to fatal prostate cancer. Cancer Epidemiol. Biomark. Prev..

[52] Park J.M., Nam J.S., Na W., Oh J.J., Lee S., Hong S.K., Byun S.S., Lee S.E. (2012). Prognostic value of body mass index in korean patients with castration-resistant prostate cancer. Korean J. Urol..

[53] Häggström C., Stocks T., Ulmert D., Bjørge T., Ulmer H., Hallmans G., Manjer J., Engeland A., Nagel G., Almqvist M. (2012). Prospective study on metabolic factors and risk of prostate cancer. Cancer.

[54] Discacciati A., Orsini N., Andersson S.O., Andrén O., Johansson J.E., Wolk A. (2011). Body mass index in early and middle-late adulthood and risk of localised, advanced and fatal prostate cancer: A population-based prospective study. Br. J. Cancer.

[55] Dehal A., Garrett T., Tedders S.H., Arroyo C., Afriyie-Gyawu E., Zhang J. (2011). Body mass index and death rate of colorectal cancer among a national cohort of U.S. adults. Nutr. Cancer.

[56] Geinitz H., Thamm R., Mueller T., Jess K., Zimmermann F.B., Molls M., Nieder C. (2011). Impact of body mass index on outcomes after conformal radiotherapy in patients with prostate cancer. Int. J. Radiat. Oncol. Biol. Phys..

[57] Van Roermund J.G., Hinnen K.A., Battermann J.J., Witjes J.A., Bosch J.L., Kiemeney L.A., van Vulpen M. (2010). Body mass index is not a prognostic marker for prostate-specific antigen failure and survival in Dutch men treated with brachytherapy. BJU Int..

[58] Han M., Trock B.J., Partin A.W., Humphreys E.B., Bivalacqua T.J., Guzzo T.J., Walsh P.C. (2010). The impact of preoperative erectile dysfunction on survival after radical prostatectomy. BJU Int..

[59] Burton A., Martin R., Galobardes B., Davey Smith G., Jeffreys M. (2010). Young adulthood body mass index and risk of cancer in later adulthood: Historical cohort study. Cancer Causes Control..

[60] Martin R.M., Vatten L., Gunnell D., Romundstad P., Nilsen T.I. (2009). Components of the metabolic syndrome and risk of prostate cancer: The HUNT 2 cohort, Norway. Cancer Causes Control..

[61] Pfitzenmaier J., Pritsch M., Haferkamp A., Jakobi H., Fritsch F., Gilfrich C., Djakovic N., Buse S., Pahernik S., Hohenfellner M. (2009). Is the body mass index a predictor of adverse outcome in prostate cancer after radical prostatectomy in a mid-European study population?. BJU Int..

[62] Davies B.J., Smaldone M.C., Sadetsky N., Dall’era M., Carroll P.R. (2009). The impact of obesity on overall and cancer specific survival in men with prostate cancer. J. Urol..

[63] Armstrong A.J., Halabi S., de Wit R., Tannock I.F., Eisenberger M. (2009). The relationship of body mass index and serum testosterone with disease outcomes in men with castration-resistant metastatic prostate cancer. Prostate Cancer Prostatic Dis..

[64] Ma J., Li H., Giovannucci E., Mucci L., Qiu W., Nguyen P.L., Gaziano J.M., Pollak M., Stampfer M.J. (2008). Prediagnostic body-mass index, plasma C-peptide concentration, and prostate cancer-specific mortality in men with prostate cancer: A long-term survival analysis. Lancet Oncol..

[65] Merrick G.S., Galbreath R.W., Butler W.M., Wallner K.E., Allen Z.A., Adamovich E. (2007). Obesity is not predictive of overall survival following permanent prostate brachytherapy. Am. J. Clin. Oncol..

[66] Efstathiou J.A., Bae K., Shipley W.U., Hanks G.E., Pilepich M.V., Sandler H.M., Smith M.R. (2007). Obesity and mortality in men with locally advanced prostate cancer: Analysis of RTOG 85-31. Cancer.

[67] Gong Z., Agalliu I., Lin D.W., Stanford J.L., Kristal A.R. (2007). Obesity is associated with increased risks of prostate cancer metastasis and death after initial cancer diagnosis in middle-aged men. Cancer.

[68] Montgomery R.B., Goldman B., Tangen C.M., Hussain M., Petrylak D.P., Page S., Klein E.A., Crawford E.D., Southwest Oncology Group (2007). Association of body mass index with response and survival in men with metastatic prostate cancer: Southwest Oncology Group trials 8894 and 9916. J. Urol..

[69] Wright M.E., Chang S.C., Schatzkin A., Albanes D., Kipnis V., Mouw T., Hurwitz P., Hollenbeck A., Leitzmann M.F. (2007). Prospective study of adiposity and weight change in relation to prostate cancer incidence and mortality. Cancer.

[70] Huxley R., Ansary-Mohaddam A., Huxley R., Barzi F., Lam T.H., Jamrozik K., Ohkubo T., Fang X., Sun H.J., Asia Pacific Cohort Studies Collaboration (2007). The impact of modifiable risk factors on mortality from prostate cancer in populations of the Asia-Pacific region. Asian Pac. J. Cancer Prev..

[71] Halabi S., Ou S.S., Vogelzang N.J., Small E.J. (2007). Inverse correlation between body mass index and clinical outcomes in men with advanced castration-recurrent prostate cancer. Cancer.

[72] Siddiqui S.A., Inman B.A., Sengupta S., Slezak J.M., Bergstralh E.J., Leibovich B.C., Zincke H., Blute M.L. (2006). Obesity and survival after radical prostatectomy: A 10-year prospective cohort study. Cancer.

[73] Park S.M., Lim M.K., Shin S.A., Yun Y.H. (2006). Impact of prediagnosis smoking, alcohol, obesity, and insulin resistance on survival in male cancer patients: National Health Insurance Corporation Study. J. Clin. Oncol..

[74] Eichholzer M., Bernasconi F., Jordan P., Stähelin H.B. (2005). Body mass index and the risk of male cancer mortality of various sites: 17-year follow-up of the Basel cohort study. Swiss Med. Wkly..

[75] Rodriguez C., Patel A.V., Calle E.E., Jacobs E.J., Chao A., Thun M.J. (2001). Body mass index, height, and prostate cancer mortality in two large cohorts of adult men in the United States. Cancer Epidemiol. Biomark. Prev..

[76] Gapstur S.M., Gann P.H., Colangelo L.A., Barron-Simpson R., Kopp P., Dyer A., Liu K. (2001). Postload plasma glucose concentration and 27-year prostate cancer mortality (United States). Cancer Causes Control..

[77] Andersson S.O., Wolk A., Bergström R., Adami H.O., Engholm G., Englund A., Nyrén O. (1997). Body size and prostate cancer: A 20-year follow-up study among 135006 Swedish construction workers. J. Natl. Cancer Inst..

[78] Huedo-Medina T.B., Sánchez-Meca J., Marín-Martínez F., Botella J. (2006). Assessing heterogeneity in meta-analysis: Q statistic or I2 index?. Psychol. Methods.

[79] Khan K.S., Ball E., Fox C.E., Meads C. (2012). Systematic reviews to evaluate causation: An overview of methods and application. Evid. Based Med..

[80] Gerdtsson A., Poon J.B., Thorek D.L., Mucci L.A., Evans M.J., Scardino P., Abrahamsson P.A., Nilsson P., Manjer J., Bjartell A. (2015). Anthropometric Measures at Multiple Times Throughout Life and Prostate Cancer Diagnosis, Metastasis, and Death. Eur. Urol..

[81] Chamberlain C., Romundstad P., Vatten L., Gunnell D., Martin R.M. (2011). The association of weight gain during adulthood with prostate cancer incidence and survival: A population-based cohort. Int. J. Cancer.

[82] Nguyen P.L., Ma J., Chavarro J.E., Freedman M.L., Lis R., Fedele G., Fiore C., Qiu W., Fiorentino M., Finn S. (2010). Fatty acid synthase polymorphisms, tumor expression, body mass index, prostate cancer risk, and survival. J. Clin. Oncol..

[83] Arcidiacono B., Iiritano S., Nocera A., Possidente K., Nevolo M.T., Ventura V., Foti D., Chiefari E., Brunetti A. (2012). Insulin resistance and cancer risk: An overview of the pathogenetic mechanisms. Exp. Diabetes Res..

[84] Santoni M., Cimadamore A., Massari F., Piva F., Aurilio G., Martignetti A., Scarpelli M., Di Nunno V., Gatto L., Battelli N. (2019). Key Role of Obesity in Genitourinary Tumors with Emphasis on Urothelial and Prostate Cancers. Cancers.

[85] Petrelli F., Cortellini A., Indini A., Tomasello G., Ghidini M., Nigro O., Salati M., Dottorini L., Iaculli A., Varricchio A. (2021). Association of Obesity With Survival Outcomes in Patients With Cancer: A Systematic Review and Meta-analysis. JAMA Netw. Open.

[86] Annett S., Moore G., Robson T. (2020). Obesity and Cancer Metastasis: Molecular and Translational Perspectives. Cancers.

[87] World Cancer Research Fund. American Institute for Cancer Research. Body Fatness and Weight Gain. https://www.wcrf.org/dietandcancer/exposures/body-fatness.

[88] Freedland S.J., Howard L., Allen J., Smith J., Stout J., Aronson W., Inman B.A., Armstrong A.J., George D., Westman E. (2019). A lifestyle intervention of weight loss via a low-carbohydrate diet plus walking to reduce metabolic disturbances caused by androgen deprivation therapy among prostate cancer patients: Carbohydrate and prostate study 1 (CAPS1) randomized controlled trial. Prostate Cancer Prostatic Dis..

[89] Schenk J.M., Neuhouser M.L., Beatty S.J., VanDoren M., Lin D.W., Porter M., Gore J.L., Gulati R., Plymate S.R., Wright J.L. (2019). Randomized trial evaluating the role of weight loss in overweight and obese men with early stage prostate Cancer on active surveillance: Rationale and design of the Prostate Cancer Active Lifestyle Study (PALS). Contemp. Clin. Trials.

