# Supplementary material for: Obesity as a Risk Factor for Prostate Cancer Mortality: A Systematic Review and Dose-Response Meta-Analysis of 280,199 Patients
Source: Cancers (Basel). 2021 Aug 19;13(16):4169. doi: 10.3390/cancers13164169 (PMC8392042; doi:10.3390/cancers13164169)
Supplement: Supplementary file 1 [file cancers-13-04169-s001.zip › Supplementary Table S2.pdf]

Supporting methods.

Search terms.

1. prostat* cancer.ti,ab

2. prostat* neoplasm.ti,ab

3. prostat* tumor.ti,ab

4. mortality.ti,ab

5. death.ti,ab

6. prognos*.ti,ab

7. survival.ti,ab

8. outcome*.ti,ab

9. obes*.ti,ab

10. body mass index.ti,ab

11. BMI.ti,ab

12. weight.ti,ab

13. cohort.ti.ab

14. case-control.ti.ab

15. follow-up.ti.ab

16. 1 OR 2 OR 3

17. 4 OR 5 OR 6 OR 7 OR 8

18. 9 OR 10 OR 11 OR 12

19. 13 OR 14 OR 15

20. 16 AND 17 AND 18 AND 19
