# Supplementary material for: Obesity as a Risk Factor for Prostate Cancer Mortality: A Systematic Review and Dose-Response Meta-Analysis of 280,199 Patients
Source: Cancers (Basel). 2021 Aug 19;13(16):4169. doi: 10.3390/cancers13164169 (PMC8392042; doi:10.3390/cancers13164169)
Supplement: Supplementary file 1 [file cancers-13-04169-s001.zip › Supplementary Table S3.pdf]

Main characteristics of the studies included in the systematic review of prognosis of prostate cancer with respect to obesity.

Author	Year	Country	Follow-up	Design	Participants and population	Exposure	Outcome	Adjustment variables	NOS
Bluethmann	2020	USA	2004-2014	cohort, prospective	90,694 PC survivors of >40 years	BMI postdiagnosis	PCSM	age, race/ethnicity, insurance status, rurality, lymph node status, PC aggressiveness, serum PSA, smoking, physical inactivity, chronic drinking, and fruit/vegetable intake	6
Crump	2020	Sweden	1972-2017, mean 39.5 years	cohort, prospective	699,125 participants. 10,782 incident PC	BMI prediagnosis	PCSM	age, year of military conscription examination, CRF, muscle strength, country of birth, education, neighbourhood SES, FHPC	9
Jochems	2020	Sweden	1971-2016, mean 28 years	cohort, prospective	431,902 participants. 38,871 incident PC	BMI prediagnosis	PCSM	age at study entry, smoking status, healthcare region, country of birth and education, income closest to diagnosis, source of income closest to diagnosis, civil status closest to diagnosis, Charlson comorbidity index, primary treatment for PC, PC risk category	8
Vidal	2020	USA	1990-2019, median 7.4 years	cohort, retrospective	5,929 PC patients who underwent RP	BMI postdiagnosis	PCSM	age, race, surgical center, year of surgery, PSA, biopsy grade group, pathologic grade group (not biopsy), positive surgical margins, seminal vesicle invasion, extracapsular invasion, and lymph node involvement	7
Jackson	2020	Jamaica	2005-2017, median 11.3 years	cohort, retrospective	242 incident PC in African-Caribbean ancestry	BMI postdiagnosis	PCSM, A-CM	age, smoking, diabetes, Gleason score, primary treatment modality, FHPC and androgen deprivation therapy	6
Troeschel	2020	USA	1992-2016, median 5.7 years	cohort, prospective	11,788 participants. 8,330 non-metastatic PC	BMI postdiagnosis	PCSM, A-CM	age, education, smoking, PSA, T, Gleason, initial treatment, year of diagnosis	6
Dickerman	2019	Iceland	2002-2015, median 10.1 years	cohort, prospective	5,764 participants. 172 incident PC	BMI postdiagnosis	PCSM	age, family FHPC, smoking status, education, frequency of moderate/ vigorous physical activity during youth and midlife, presence of a physician visit over the past year	8
Langlais	2019	USA	1995-2018, median 4.5 years	cohort, prospective	3,230 PC patients who underwent RP	BMI postdiagnosis	A-CM	age, race, smoking status, comorbidities, surgical approach, PSA, pathologic Gleason	6

								score, pathologic T-stage pathologic N-stage, and clinical stage	
Darcey	2019	Australia	2001-2017, median 15 years	cohort, retrospective	572 incident PC	BMI postdiagnosis	PCSM, A-CM	age, Gleason score	7
Wade	2019	Sweden	1961-2004	cohort, prospective	996,898 participants, 12,712 incident PC	BMI prediagnosis	PCSM	educational and occupation socioeconomical index	8
Farris	2018	Canada	1997-2017	cohort, prospective	829 incident PC	BMI postdiagnosis	PCSM, A-CM	age at diagnosis, stage, prostatectomy, initial hormone therapy treatment, initial radiation therapy treatment, PSA levels at diagnosis, and post-diagnosis Charlson comorbidity index score	7
Hu	2018	China	2003-2015, median 49 months	cohort, retrospective	435 PC patients receiving androgen deprivation therapy	BMI postdiagnosis	A-CM	age, diabetes, Japan Cancer of the Prostate Risk Assessment (J-CAPRA) score	6
Pérez-Cornago	2017	Europe	1992-2013, average 13.9 years	cohort, prospective	141,896 participants, 7,024 incident PC	BMI postdiagnosis	PCSM	age, center, education level, smoking status, marital status, Diabetes, physical activity	9
Dickerman	2017	USA	1986-2012,	cohort, prospective	5,158 patients with clinically localized PC	BMI postdiagnosis	PCSM	age at diagnosis, race, FHPC, smoking status at diagnosis, diabetes at diagnosis, heart/lung disease by time of diagnosis, physical activity, energy intake, tomato sauce intake, coffee intake, alpha-linolenic acid and calcium intake, clinical stage, grade, PSA at diagnosis, primary treatment	7
Kelly	2017	USA	1993-2009, median 11.5 years	cohort, prospective	7,822 incident PC	BMI prediagnosis	PCSM	screening arm, family history of prostate cancer, race, study center, education, marital status, cigarette smoking status, diabetes, myocardial infarction, PSA history during the three years prior to enrolment	8
Polesel	2016	Italy	1995-2002, median 11.6 years	cohort, retrospective	715 incident PC	BMI prediagnosis	PCSM, A-CM	age, area of residence, calendar period, years of education, Gleason score, smoking habits	7
Cushen	2016	Ireland	2008-2014	cohort, retrospective	63 patients with metastatic castrate-resistant PC treated with docetaxel	BMI postdiagnosis	A-CM	age, bone metastases and non-osseous metastases, anaemia, muscle density, sarcopenia, visceral fat (VAT) index and subcutaneous fat (SAT) index	5

Fowke	2015	Asian countries	1963-2006, mean 9 years	18 cohort, prospective studies	522,736 participants, 634 PC deaths	BMI prediagnosis	PCSM	age, education, population density, marital status, history of severe cancer, heart disease, or stroke at baseline	7
Wang	2015	USA	2001-2010, median 47.6 months	cohort, retrospective	1,442 localized PC after receiving intensity-modulated RT	BMI postdiagnosis	PCSM, A-CM	age, androgen deprivation therapy (ADT), pretreatment PSA as a log-transformed variable (iPSA), Gleason score and T classification	8
Cantarutti	2015	Sweden	2001-2012	cohort, retrospective	3,161 incident PC	BMI prediagnosis	PCSM, A-CM	age at inclusion to study, PSA, treatment	7
Taghizadeh	2015	The Netherlands	1965-2008, median 34.5 years	cohort, prospective	3,718 incident PC	BMI prediagnosis	PCSM	age, smoking and place of residence	7
Mohammed	2015	Saudi Arabia	2011-2015, median 14.4 months	cohort, retrospective	71 metastatic PC	BMI postdiagnosis	PCSM	age, PSA, alkaline phosphatase, visceral metastasis, bone, lactate dehydrogenase, albumin and poorly differentiated pathology	7
Moller	2015	Denmark	1993-2011, median 15.5 years	cohort, prospective	26,877 participants, 1,813 incident PC	BMI prediagnosis	PCSM	stage (TNM)	7
Bonn	2014	Sweden	1997-2007, median 4 years	cohort, retrospective	4,367 clinically localized PC	BMI postdiagnosis	PCSM, A-CM	age at diagnosis, primary treatment, Gleason score at diagnosis, PSA level, T-, N- and M-stages at diagnosis, smoking habits, and total metabolic equivalent time (MET-h) at age 50	6
Chalfin	2014	USA	1982-2012, median 5 years	cohort, retrospective	11,152 PC patients who underwent RP	BMI postdiagnosis	PCSM, A-CM	age, year of surgery, race, preoperative PSA, pathology stage, pathologic Gleason sum, and positive surgical margin	6
Haque	2014	USA	1971-2001	case-control, retrospective	751 PC patients who underwent RP	BMI postdiagnosis	PCSM	age at diagnosis, health plan site, race, year of diagnosis, stage at diagnosis, duration of health plan membership, PSA at diagnosis, surgical margins status, and lymph node status	8
Froehner	2014	Germany	1992-2005, median 8.6 years	cohort, retrospective	2,131 PC patients who underwent RP	BMI postdiagnosis	A-CM	age, Gleason score, tumor stage, Charlson score and American Society of Anesthesiologists physical status class	8
Gravis	2014	France, Belgium	2004-2008, median 58.4 months	cohort, prospective	385 non-castrate metastatic PC	BMI postdiagnosis	A-CM	univariate	4

Tendulkar	2013	USA	1996-2009, median 74 months	cohort, retrospective	660 high-risk PC treated with RT and AD	BMI postdiagnosis	PCSM	univariate	3
Bassett	2012	Australia	1990-2009, mean 15 years	cohort, prospective	16,514 participants, 1,374 incident PC	BMI prediagnosis	PCSM	country of birth and education	8
Schwartz	2012	USA	1988-2006	cohort, prospective	6,707 participants, 49 PC deaths	BMI prediagnosis	PCSM	age, ionized calcium, serum albumin and 25-OH vitaminD	8
Park	2012	Korea	2003-2009, median 24.6 months	cohort, retrospective	55 castration-resistant PC	BMI postdiagnosis	PCSM	age, Gleason score, log PSA, hemoglobin, Eastern Cooperative Oncology Group Performance Status, alkaline phosphatase, visceral metastasis,prior retropublicradical prostatectomy and prior radiation therapy	7
Haggstrom	2012	Norway, Sweden, Austria	1972-2006, mean 12 years	cohort, prospective (7 cohorts)	289,866 participant, 6,673 incident PC	BMI prediagnosis	PCSM	smoking, five birth cohorts and five categories of age at measurement	8
Discaciatti	2011	Sweden	1998-2007	cohort, prospective	36,959 participants, 2,084 incident PC	BMI prediagnosis	PCSM	age at baseline, BMI at age 30 years, total energy intake, total physical activity, years of education, smoking status, family history of prostate cancer and, personal history of diabetes	7
Dehal	2011	USA	1971-1992, average 17 years	cohort, prospective	7,016 participant, 3,127 incident PC	BMI prediagnosis	PCSM	age, socio-economic status variables, cigarette smoking, alcohol drinking and dietary pattern	9
Geinitz	2011	Germany	1994-2002, median 51 months	cohort, retrospective	564 localized PC after conformal RT	BMI postdiagnosis	PCSM, A-CM	age, PSA, and radiation dose, endocrine treatment, T stage, and histological grade	7
Van Roermund	2010	The Netherlands	1991-2008, median 47 months	cohort, retrospective	1,530 localized PC who underwent brachytherapy	BMI postdiagnosis	PCSM, A-CM	age, preoperative PSA, pathological stage, pathological grade, risk group, prostate volume, preoperative pelvic lymph node dissection, treatment period and number of seeds	7
Han	2010	USA	1983-2000, median 13 years	cohort, retrospective	2,718 PC patients who underwent RP	BMI postdiagnosis	A-CM	univariate	7
Burton	2010	UK	1948-2009, median 49 years	cohort, prospective	9,549 participants, 211 incident PC	BMI prediagnosis	PCSM	smoking, father's social class and height	7

Martin	2009	Norway	1995-2005, median 9.3 years	cohort, prospective	29,364 participants, 687 incident PC	BMI prediagnosis	PCSM	age, height, smoking, marital status, education, physical activity, International Prostate Symptom Score	8
Pfitzenmaier	2009	Germany	median 5.5 years	cohort, prospective	620 PC patients who underwent RP	BMI postdiagnosis	A-CM	tumor extension, lymph node, tumor grading, PSA and resection margin	4
Davies	2009	USA	1995-2007, mean 51.3 months	cohort, prospective	7,274 clinically localized PC	BMI postdiagnosis	PCSM, A-CM	age, clinical risk, presence of diabetes and type of therapy	6
Armstrong	2009	USA	2000-2006	cohort, prospective	1,001 castration-resistant metastatic PC	BMI postdiagnosis	A-CM	baseline pain, presence of liver metastases, performance status, hemoglobin, alkaline phosphatase, PSA, PSA doubling time, number of sites of metastatic disease, type of progression, high-grade disease and treatment group	5
Ma	2008	USA	1982-2007, median 7 years	cohort, prospective	2,546 incident PC	BMI prediagnosis	A-CM	age at diagnosis, baseline smoking status, time interval from BMI measurement to PC diagnosis, clinical stage and Gleason grade	7
Merrick	2007	USA	1995-2003, mean 5.9 years	cohort, prospective	1,093 localized PC who underwent brachytherapy	BMI prediagnosis	PCSM, A-CM	univariate	5
Efstathiou	2007	USA	1987-2005, median 8.1 years	cohort, prospective	945 locally advanced PC	BMI postdiagnosis	PCSM, A-CM	age, race, treatment arm, history of prostatectomy, nodal involvement, Gleason score and clinical stage	6
Gong	2007	USA	1993-2004, average 9.5 years	cohort, prospective	752 incident PC	BMI prediagnosis	PCSM, A-CM	age at diagnosis, race, smoking status, Gleason score, stage at diagnosis and primary treatment	8
Montgomery (8894)	2007	USA	1989-1994	cohort, prospective	1,006 metastatic PC treated with AD	BMI postdiagnosis	A-CM	treatment arm, race, pre-study Gleason sum, pre-study bone pain, disease extent, performance status and pre-study PSA	5
Montgomery (9916)	2007	USA	1999-2003	cohort, prospective	671 metastatic PC treated with chemotherapy	BMI postdiagnosis	A-CM	treatment arm, race, pre-study Gleason sum, pre-study bone pain, disease extent, performance status and pre-study PSA	5
Wright	2007	USA	1995-2001	cohort, prospective	287,760 participants, 9,986 incident PC	BMI prediagnosis	PCSM	age, race, smoking status, education, personal history of diabetes, FHPC	6
Huxley	2007	Asia-Pacific region	median 6.8 years	cohort prospective, 24 cohorts	320,852 participants, 308 PC deaths	BMI prediagnosis	PCSM	smoking and diabetes	6
Halabi	2007	USA	1991-2004, median	cohort, retrospective	1,226 castration-recurrent PC	BMI posdiagnosis	PCSM, A-CM	age race, ECOG performance status, Gleason score, hemoglobin, testosterone,	6

			33.8 months					PSA, alkaline phosphatase, LDH, presence of visceral disease, prior treatment with radiotherapy, years since diagnosis	
Siddiqui	2006	USA	1990-1999, median 10.1 years	cohort, prospective	5,313 PC patients who underwent RP	BMI posdiagnosis	PCSM, A-CM	Gleason score, preoperative serum PSA, surgical margin status, seminal vesicle invasion and adjuvant treatment	6
Park	2006	Korea	1996-2004, median 3.86 years	cohort, prospective	14,578 participants, 256 incident PC	BMI prediagnosis	A-CM	age, alcohol consumption, fasting serum glucose level, cholesterol level, physical activity, food preference, blood pressure, and other comorbidities	7
Eichholzer	2005	Switzerland	1971-1990	cohort, prospective	2,974 participants, 30 PC deaths	BMI prediagnosis	PCSM	age and smoking	6
Rodriguez (CPS-I)	2001	USA	1959-1972	cohort, prospective	456,490 participants, 2,277 PC deaths	BMI prediagnosis	PCSM	age at interview, race, height, education, exercise, smoking status and FHPC	7
Rodriguez (CPS-II)	2001	USA	1982-1996	cohort, prospective	508,351 participants, 5,414 PC deaths	BMI prediagnosis	PCSM	age at interview, race, height, education, exercise, smoking status and FHPC	7
Gapstur	2001	USA	1967-1997, mean 27 years	cohort, prospective	20,433 participant, 176 PC deaths	BMI prediagnosis	PCSM	age, postload plasma glucose level, heart rate, education and race	7
Andersson	1997	Sweden	1971-1991, average 18 years	cohort, retrospective	135,006 participants, 2,368 incident PC	BMI prediagnosis	PCSM	age	7
