# Supplementary material for: Obesity as a Risk Factor for Prostate Cancer Mortality: A Systematic Review and Dose-Response Meta-Analysis of 280,199 Patients
Source: Cancers (Basel). 2021 Aug 19;13(16):4169. doi: 10.3390/cancers13164169 (PMC8392042; doi:10.3390/cancers13164169)
Supplement: Supplementary file 1 [file cancers-13-04169-s001.zip › Supplementary Table S4.pdf]

Methodological quality assessment of the selected studies according to Newcastle-Ottawa Scale.

A. Cohort studies (n=58)

		**Selection**				**Comparability**		**Outcome**			
Study	Year	Representativeness of exposed cohort	Representativeness of unexposed cohort	Ascertainment of exposure[1]	Outcome was not present at start	Important Factor[2]	Additional factor[3]	Assessment	Exposure follow-up long enough for outcomes to occur	Adequacy of follow-up[4]	**Total score**
Bluethmann	2020	0	1	0	1	1	1	1	1	0	**6**
Crump	2020	1	1	1	1	1	1	1	1	1	**9**
Jackson	2020	0	1	0	1	1	1	1	1	0	**6**
Jochems	2020	1	1	1	1	1	1	1	1	0	**8**
Vidal	2020	0	1	1	1	1	1	1	1	0	**7**
Troeschel	2020	0	1	0	1	1	1	1	1	0	**6**
Dickerman	2019	1	1	1	1	1	1	1	1	0	**8**
Langlais	2019	0	1	0	1	1	1	1	1	0	**7**
Darcey	2019	1	1	0	1	1	1	1	1	0	**7**
Wade	2019	1	1	1	1	1	1	1	1	0	**8**
Farris	2018	1	1	1	1	1	1	1	0	0	**7**
Hu	2018	0	1	1	1	1	1	1	0	0	**6**

		Selection				Comparability		Outcome			
Study	Year	Representativeness of exposed cohort	Representativeness of unexposed cohort	Ascertainment of exposure[1]	Outcome was not present at start	Important Factor[2]	Additional factor[3]	Assessment	Exposure follow-up long enough for outcomes to occur	Adequacy of follow-up[4]	**Total score**
Pérez-Cornago	2017	1	1	1	1	1	1	1	1	1	**9**
Dickerman	2017	0	1	0	1	1	1	1	1	1	**7**
Kelly	2017	1	1	0	1	1	1	1	1	1	**8**
Polesel	2016	1	1	0	1	1	1	1	1	0	**7**
Cushen	2016	0	1	0	1	1	1	1	0	0	**5**
Fowke	2015	1	1	0	1	1	1	1	1	0	**7**
Wang	2015	0	1	1	1	1	1	1	1	1	**8**
Cantarutti	2015	1	1	0	1	1	1	1	1	0	**7**
Taghizadeh	2015	1	1	0	1	1	1	0	1	1	**7**
Mohammed	2015	0	1	1	1	1	1	1	0	1	**7**
Moller	2015	1	1	1	1	0	1	1	1	0	**7**
Bonn	2014	0	1	0	1	1	1	1	1	0	**6**
Chalfin	2014	0	1	0	1	1	1	1	1	0	**6**
Froehner	2014	0	1	1	1	1	1	1	1	1	**8**
Gravis	2014	0	1	1	1	0	0	0	1	0	**4**
Tendulkar	2013	0	1	0	1	0	0	0	1	0	**3**

		**Selection**				**Comparability**		**Outcome**			
Study	Year	Representativeness of exposed cohort	Representativeness of unexposed cohort	Ascertainment of exposure[1]	Outcome was not present at start	Important Factor[2]	Additional factor[3]	Assessment	Exposure follow-up long enough for outcomes to occur	Adequacy of follow-up[4]	**Total score**
Bassett	2012	1	1	1	1	0	1	1	1	1	**8**
Schwartz	2012	1	1	1	1	1	1	1	1	0	**8**
Park	2012	0	1	1	1	1	1	0	1	1	**7**
Haggstrom	2012	1	1	1	1	1	1	1	1	0	**8**
Discaciatti	2011	1	1	0	1	1	1	1	1	0	**7**
Dehal	2011	1	1	1	1	1	1	1	1	1	**9**
Geinitz	2011	0	1	1	1	1	1	1	1	0	**7**
Van Roermund	2010	0	1	1	1	1	1	1	0	1	**7**
Han	2010	0	1	1	1	1	1	1	1	0	**7**
Burton	2010	1	1	1	1	0	1	1	1	0	**7**
Martin	2009	1	1	1	1	1	1	1	1	0	**8**
Pfitzenmaier	2009	0	1	0	1	0	1	0	1	0	**4**
Davies	2009	0	1	1	1	1	1	1	0	0	**6**
Armstrong	2009	0	1	1	1	0	1	1	0	0	**5**
Ma	2008	1	1	0	1	0	1	1	1	1	**7**
Merrick	2007	0	1	1	1	0	0	1	1	0	**5**

		Selection				Comparability		Outcome			
Efstathiou	2007	0	1	1	1	1	1	0	1	0	**6**
		**Selection**				**Comparability**		**Outcome**			
		Representativeness of exposed cohort	Representativeness of unexposed cohort	Ascertainment of exposure[1]	Outcome was not present at start	Important Factor[2]	Additional factor[3]	Assessment	Exposure follow-up long enough for outcomes to occur	Adequacy of follow-up[4]	**Total score**
Gong	2007	1	1	0	1	1	1	1	1	1	**8**
Montgomery (8894)	2007	0	1	1	1	0	1	0	0	1	**5**
Montgomery (9916)	2007	0	1	1	1	0	1	0	0	1	**5**
Wright	2007	1	1	0	1	1	1	1	0	0	**6**
Huxley	2007	1	1	1	1	0	1	0	1	0	**6**
Halabi	2007	0	1	0	1	1	1	0	1	1	**6**
Siddiqui	2006	0	1	1	1	0	1	0	1	1	**6**
Park	2006	1	1	1	1	1	1	1	0	0	**7**
Eichholzer	2005	0	1	1	1	1	1	0	1	0	**6**
Rodriguez (CPS-I)	2001	1	1	0	1	1	1	0	1	1	**6**
Rodriguez (CPS-II)	2001	1	1	0	1	1	1	0	1	1	**6**
Gapstur	2001	0	1	1	1	1	1	1	1	0	**7**
Andersson	1997	0	1	1	1	1	0	1	1	1	**7**

[1] The point was assigned for objectively measured body mass index.
[2] If adjusted for age, the point was assigned.

[3] If adjusted for any other additional factor, the point was assigned.
[4] If the completeness of follow-up was 90% or more, or characteristics of lost cohort were similar to the rest of the cohort, the point was assigned.

B. Case-control studies (n = 1)

		**Selection**				**Comparability**		**Exposure**			
Study	Year	Definition of cases	Representativeness of cases	Selection of controls	Definition of controls	Important Factor[1]	Additional factor[2]	Ascertainment[3]	Same method for subjects	Non-response rate	Total score
Haque	2014	1	1	1	1	1	1	0	1	1	**8**

[1] If adjusted for age, the point was assigned.
[2] If adjusted for any other additional factor, the point was assigned.
[3] The point was assigned for objectively measured body mass index.
