# Supplementary material for: Obesity as a Risk Factor for Prostate Cancer Mortality: A Systematic Review and Dose-Response Meta-Analysis of 280,199 Patients
Source: Cancers (Basel). 2021 Aug 19;13(16):4169. doi: 10.3390/cancers13164169 (PMC8392042; doi:10.3390/cancers13164169)
Supplement: Supplementary file 1 [file cancers-13-04169-s001.zip › Supplementary Table S5.pdf]

Assessment of potential causation criteria for the association assessed in this systematic review.

**Causal criteria**	**Evaluative systematic review methods applied**
Strength of association	The measures of strength of association in this systematic review were hazard ratios (HR) for quantifying the association between obesity and mortality of each individual study. Pooled analysis showed HR = 1.19 (95% CI: 1.10-1.28) for the association between obesity and prostate cancer specific mortality and HR = 1.09 (95% CI: 1.00-1.18) for the association between obesity and all-cause mortality. The strength of association between obesity and prostate cancer specific mortality was especially high when analysing the subgroup of higher quality (HR = 1.24, 95% CI: 1.14-1.25).
Consistency	Consistency of individual studies was analysed graphically using forest plots and statistically by χ^2 test and I^2 statistic. We performed heterogeneity analysis to evaluate sources of heterogeneity and obtained pooled results with acceptable heterogeneity (several subgroups showed I^2=0.0%). When analysing the pooled estimators stratified by potential heterogeneity factors, the associations between obesity and prostate cancer specific or all-cause mortality remained.
Temporality	We only included follow-up (cohort or case-control) studies. Cross-sectional studies were excluded from the search. We also evaluated if the outcome was proven to be absent at the beginning of the study to ensure temporality. This item was evaluated in the Newcastle-Ottawa Scale (NOS). The pooled results according to NOS (high quality) and according to design (prospective cohort studies) showed the same associations.
Specificity	Attempts to study the specifical effect of obesity on mortality were made through heterogeneity analyses.
Biological gradient	We analysed the gradient of BMI on mortality and showed greater association according to higher BMI strata. Continuous BMI per 5 kg/m^2 were analysed in the dose-response meta-analysis association were found regarding prostate cancer specific and all-cause mortality.
Plausibility, coherence and analogy	Obesity is presumed aetiological factor biologically plausible. There are also analogous causal relationships between the aetiological factor and mortality from other cancers. These conditions have been analysed throughout the discussion section of the manuscript.
Experimental evidence	Not applicable to risk factors.
