# Supplementary material for: Obesity as a Risk Factor for Prostate Cancer Mortality: A Systematic Review and Dose-Response Meta-Analysis of 280,199 Patients
Source: Cancers (Basel). 2021 Aug 19;13(16):4169. doi: 10.3390/cancers13164169 (PMC8392042; doi:10.3390/cancers13164169)
Supplement: Supplementary file 1 [file cancers-13-04169-s001.zip › Supplementary Table S6.pdf]

Forest-plot of the associations between obesity (BMI ≥ 30), overweight (BMI < 30 and ≥ 25) and abnormal weight (BMI ≥ 25), compared to normal weight (BMI < 25), and continuous BMI per 5 units, on prostate cancer specific mortality and all-cause mortality.

	Studies (n)	Events (n)	Participants (n)	Hazard ratio (95% CI)	p value *	I^2 (%)
**Prostate Cancer specific mortality**						
BMI continuous, per 5 kg/m^2	31	30,891	148,786	1.09 (1.05–1.12)	0.003	44,3
Overweight (BMI ≥ 25 & < 30)	36	13,621	154,585	1.05 (0.99–1.11)	0.003	44,3
Abnormal weight (BMI ≥ 25)	33	19,637	133,450	1.07 (0.96–1.19)	<0.001	83,6
Obesity (BMI ≥ 30)	37	20,721	245,279	1.19 (1.10–1.28)	0.002	44,4
**All-cause mortality**						
BMI continuous, per 5 kg/m^2	10	5,795	29,959	1.03 (1.01–1.05)	0.220	24,3
Overweight (BMI ≥ 25 & < 30)	21	10,849	51,161	0.93 (0.88–0.98)	0.146	24,9
Abnormal weight (BMI ≥ 25)	23	10,818	50,847	0.95 (0.86–1.03)	<0.001	74,2
Obesity (BMI ≥ 30)	23	11,302	53,677	1.09 (1.00–1.18)	0.013	43,9

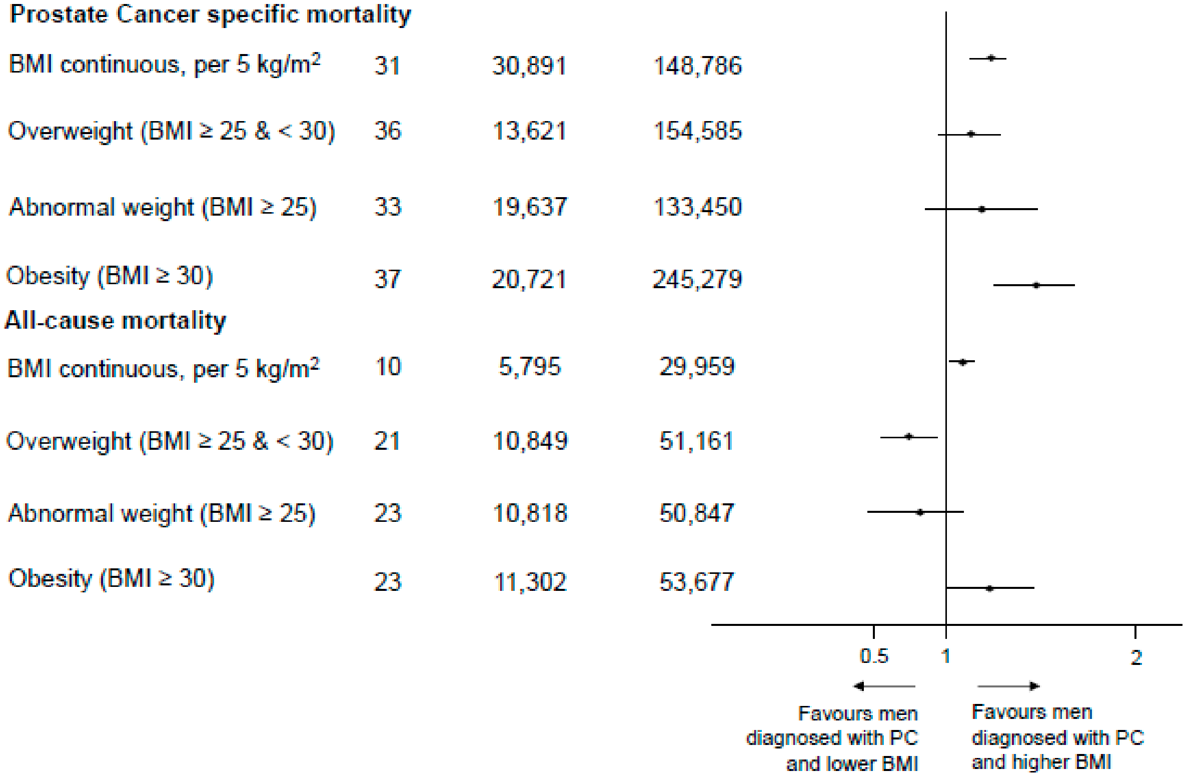

** P-values of the heterogeneity test.*
