# Supplementary material for: Obesity as a Risk Factor for Prostate Cancer Mortality: A Systematic Review and Dose-Response Meta-Analysis of 280,199 Patients
Source: Cancers (Basel). 2021 Aug 19;13(16):4169. doi: 10.3390/cancers13164169 (PMC8392042; doi:10.3390/cancers13164169)

Funnel plots of the pooled associations between obesity (BMI ≥ 30) and continuous BMI per 5 kg/m^2 with prostate cancer specific mortality and all-cause mortality.

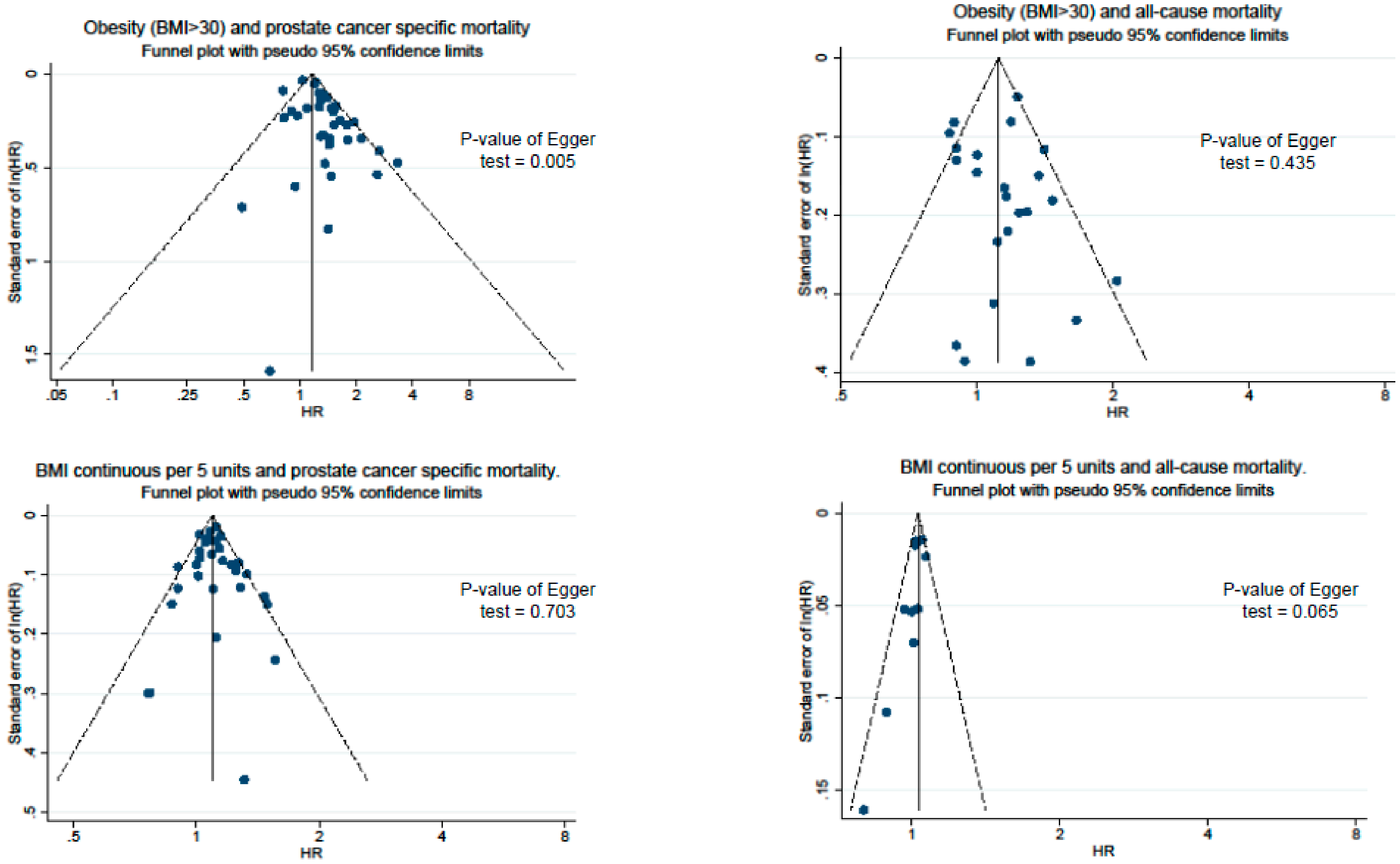

Supplement: Supplementary file 1 [file cancers-13-04169-s001.zip › Supplementary Table S7.pdf]
